# Whole Genome Multi-Locus Sequence Typing and Genomic Single Nucleotide Polymorphism Analysis for Epidemiological Typing of *Pseudomonas aeruginosa* From Indonesian Intensive Care Units

**DOI:** 10.3389/fmicb.2022.861222

**Published:** 2022-07-14

**Authors:** Manisha Goyal, Andreu Coello Pelegrin, Magali Jaillard, Yulia Rosa Saharman, Corné H. W. Klaassen, Henri A. Verbrugh, Juliëtte A. Severin, Alex van Belkum

**Affiliations:** ^1^bioMérieux Open Innovation and Partnerships, Macry-LÉtoile, France; ^2^bioMérieux EU Data Science, Macry-LÉtoile, France; ^3^Department of Clinical Microbiology, Faculty of Medicine, Dr. Cipto Mangunkusumo General Hospital, Universitas Indonesia, Jakarta, Indonesia; ^4^Department of Medical Microbiology and Infectious Diseases, Erasmus MC University Medical Center, Rotterdam, Netherlands

**Keywords:** *Pseudomonas aeruginosa*, genome sequencing, wgMLST, wgSNPs, virulome, resistome

## Abstract

We have previously studied carbapenem non-susceptible *Pseudomonas aeruginosa* (CNPA) strains from intensive care units (ICUs) in a referral hospital in Jakarta, Indonesia (Pelegrin et al., 2019). We documented that CNPA transmissions and acquisitions among patients were variable over time and that these were not significantly reduced by a set of infection control measures. Three high risk international CNPA clones (sequence type (ST)235, ST823, ST357) dominated, and carbapenem resistance was due to carbapenemase-encoding genes and mutations in the porin OprD. Pelegrin et al. (2019) reported core genome analysis of these strains. We present a more refined and detailed whole genome-based analysis of major clones represented in the same dataset. As per our knowledge, this is the first study reporting Single Nucleotide Polymorphisms (wgSNP) analysis of *Pseudomonas* strains. With whole genome-based Multi Locus Sequence Typing (wgMLST) of the 3 CNPA clones (ST235, ST357 and ST823), three to eleven subgroups with up to 200 allelic variants were observed for each of the CNPA clones. Furthermore, we analyzed these CNPA clone clusters for the presence of wgSNP to redefine CNPA transmission events during hospitalization. A maximum number 35350 SNPs (including non-informative wgSNPs) and 398 SNPs (ST-specific_informative-wgSNPs) were found in ST235, 34,570 SNPs (including non-informative wgSNPs) and 111 SNPs (ST-specific_informative-wgSNPs) in ST357 and 26,443 SNPs (including non-informative SNPs) and 61 SNPs (ST-specific_informative-wgSNPs) in ST823. ST-specific_Informative-wgSNPs were commonly noticed in sensor-response regulator genes. However, the majority of non-informative wgSNPs was found in conserved hypothetical proteins or in uncharacterized proteins. Of note, antibiotic resistance and virulence genes segregated according to the wgSNP analyses. A total of 8 transmission chains for ST235 strains followed by 9 and 4 possible transmission chains for ST357 and ST823 were traceable on the basis of pairwise distances of informative-wgSNPs (0 to 4 SNPs) among the strains. The present study demonstrates the value of detailed whole genome sequence analysis for highly refined epidemiological analysis of *P. aeruginosa*.

## Introduction

*Pseudomonas aeruginosa* is a metabolically versatile Gram-negative bacterial species often blooming in soil and aquatic environments. It effectively colonizes the exposed surfaces of plants, animals and humans (Kerr and Snelling, 2009; Klockgether and Tummler, 2017). Being an opportunistic pathogen, *P. aeruginosa* is responsible for a broad spectrum of acute and chronic infections leading to high morbidity and mortality rates (Bedard et al., 2016; Juan et al., 2017; Jacobs et al., 2020). *P. aeruginosa* causes, among others, bloodstream infections in immunocompromised patients and healthcare-associated infections such as ventilator-associated pneumonia and wound infections (Lodise et al., 2007; Kerr and Snelling, 2009; Doring et al., 2011). In the United States, *P. aeruginosa* causes a total of about 51,000 deadly healthcare infections per year (Fujii et al., 2014; CDC, 2019). Moreover, *P. aeruginosa* is known for its potential multidrug resistance (MDR) and has become one of the most troublesome causes of a wide range of intensive care unit (ICU)-acquired infections (Moore et al., 2014; Pelegrin et al., 2021). The ability to develop antibiotic resistance *via* both mutations and resistance gene acquisitions renders *P. aeruginosa* an increasingly problematic human pathogen (Livermore, 2002; Cabot et al., 2016; De Oliveira et al., 2020). Mutations that cause antibiotic impermeability *via* the loss of OprD transmembrane channels are important in antimicrobial resistance (AMR) to carbapenems (AE Studemeister and Quinn, 1988; Livermore, 2002; Puja et al., 2020; Suresh et al., 2020). MDR isolates require careful epidemiological tracing, both locally, nationally and globally.

Microbiological epidemiology defines patterns of distribution for pathogens such as *P. aeruginosa.* It also precisely assesses spreading of infectious diseases in a variety of populations (Gad, 2014). In practice, microbiological epidemiological analysis often begins with microbial strain characterization. Multi Locus Sequence Typing (MLST) is a commonly used classical approach for *P. aeruginosa* strain characterization, it accurately defines evolutionary descent and lineages but it lacks the necessary resolution for the precise characterization of outbreaks caused by closely related, contemporaneous bacterial isolates (Inns et al., 2015; Ashton et al., 2016). Several studies have evaluated the discriminatory power and concordance of different typing methods (Rumore et al., 2018; Gateau et al., 2019). However, high throughput whole genome sequencing (WGS) is rapidly becoming the most efficient solution for strain typing of *P. aeruginosa*, both for surveillance as well as for (retrospective) outbreak investigations (Kan et al., 2018). WGS facilitates whole genome MLST (wgMLST) which displays higher discrimination than conventional MLST which is based on the analysis of seven housekeeping genes only. wgMLST reliably recognizes and quantifies the genetic links between epidemiologically related isolates within various bacterial species (Cody et al., 2013; Joensen et al., 2014; Kovanen et al., 2014). A recent study (Blanc et al., 2020) showed that the *P. aeruginosa* wgMLST scheme in BioNumerics™ is as discriminatory as the core genome Single Nucleotide Polymorphism (cgSNP) calling approach and is hence useful for outbreak investigations. Whole genome SNP analysis (wgSNP) is a more advanced method of exploiting variation at the WGS level to help identify bacterial transmission dynamics and to generate useful insights into the sources and routes of infection, again for essentially all bacterial species assuming that there is a sufficient number of diverse genome sequences available (Bakker et al., 2011; Halachev et al., 2014; Taylor et al., 2015).

In a prior study, relatedness of carbapenem non-susceptible *P. aeruginosa* (CNPA) strains from an Indonesian hospital was analyzed at the cgSNP level (Pelegrin et al., 2019). In the present study, epidemiological correlation between the same isolates is studied on the basis of wgMLST and wgSNP analyses. Detailed wgSNP analysis was done for the pandemic *P. aeruginosa* sequence types (ST) ST235, ST357 and ST823 to reveal exact transmission patterns among patients and between patients, and the environment.

## Methodology

### Strain Collection

We have used preexisting genomic data of CNPA strains collected in two ICUs of a large referral hospital in Jakarta, Indonesia (see the dataset used by Pelegrin et al., 2019; Saharman et al., 2019). For each patient involved the dates of admission and discharge from the ICU were available, as well as the date of all cultures taken during ICU stay. All patients were screened for CNPA on admission, at discharge and weekly if their stay exceeded 7 days. Patients were additionally sampled upon clinical indication. Patients were enrolled in two separate episodes, before and after an infection prevention and control intervention. In the pre-intervention period ICU personnel was screened once and the ICU environment was screened twice.

Sequencing was done for clinical isolates deriving from the samples of pre-intervention phase using either a HiSeq 2500 instrument (Illumina Inc., Cambridge, United Kingdom) with 150-bp paired-end reads or a MiSeq instrument (Illumina Inc.) with 200-bp paired-end reads. Strains from the post-intervention phase were sequenced using a NextSeq 500 instrument (Illumina Inc.), with 150-bp paired-end reads. Reads and assemblies from all sequenced samples are available at the European Nucleotide Archive website under project identifiers (IDs) PRJEB30625 and PRJEB32907, for the clinical and environmental samples, respectively. From most of the patient multiple serial isolates were collected in this study. Each patient was named as a unique ID number such as 6 or 222 etc. Since multiple isolates were collected form single patient, the alphabets were allotted along with the patients unique numeric ID in order to identify each collected isolate independently irrespective of their source of collection. Patients who were colonized by multiple strains were considered while tracing the transmission events. For example isolates from patients ID 6 and 222 isolate ID 6B and 222A, respectively, can be seen in ST235 as well as in ST357 (isolate ID 6A and 222B). For the present study all CNPA genome sequences were assembled and analyzed using BioNumerics™ (Applied Maths, bioMérieux, Belgium; Supplementary Material). Antibiotic susceptibility testing (AST) of CNPA strains was performed as described by Pelegrin et al. (2019) using VITEK2 (bioMérieux).

### MLST and wgMLST Analysis

Classical MLST typing is based on polymorphisms in seven housekeeping genes stored in the *P. aeruginosa* pubMLST database.^1^ Although MLST analysis for CNPA was already published previously (Pelegrin et al., 2019), here we have repeated the analysis with an updated version of the pubMLST database in order to compare up-to-date MLST with the current wgMLST analysis. wgMLST typing of 237 CNPA genomes was performed using BioNumerics™. For wgMLST typing, fully functional and well curated schemes have been developed and maintained for many important pathogens including *P. aeruginosa* in BioNumerics™ plugins.^2^ A total of 15,143 genes and other genetic elements were used to assign wgMLST types to the isolates in the CNPA collection. Allelic differences between isolates sharing the same MLST group were calculated and sub-groupings within the MLST groups were visualized as UPGMA based phylogenetic trees.

### wgSNP Analysis

Using the BioNumerics™ wgSNP application^3^ wgSNPs were identified and mapped on CNPA genomes using the *P. aeruginosa* reference genome PAO-1 (Stover et al., 2000) and NCBI Reference Sequence: NC_002516.2 (Pelegrin et al., 2019; Subedi et al., 2019). Functional annotation was performed for each SNP. The option of SNP filtering was chosen during the analysis in order to remove ambiguous bases, unreliable bases and gaps (Pongpanich et al., 2010; Kumar et al., 2012). This filter removes SNPs at positions where at least one isolate in the analysis has an unreliable base that has a very low quality scores. This result in insufficient confidence to call the base for that position. However, ambiguous base filter remove positions with at least one mixed base, which could be due to sequencing errors or assembly errors, mixed infection. Therefore because of the presence of a mixed base, isolate cannot be designated as either matching the reference or having the SNP which has implications for where the isolate is mapped on the phylogenetic tree. Because of this filtering, the number of wgSNPs dropped from many thousands to about 3,000–4,000 which still includes non-informative wgSNPs. Since the non-informative wgSNPs which are present in all the isolates when matched with a reference, they do not provide any information about the genetic relationship among the isolates. Therefore, non-informative SNP filtering (also called strict filtering) was used to select only informative-wgSNPs which were under a few 100 in number per genome within each major ST where we call it ST_specific-informative-wgSNPs. Phylogenetic tree was built on the basis of informative-wgSNPs and correlation studies were performed to identify the links informative-wgSNPs, wgMLST, patient characteristics, sample type, and the resistomes and virulomes of each CNPA isolate. Resistomes and virulomes were defined using the command line script of Torsten Seemann called Abricate and which is available at Github^4^ (Zankari et al., 2012; Chen et al., 2016). Moreover, detailed maps of functional point mutations in the three most prominent classical MLST groups (ST823, ST235 and ST357) were made and possible transmission chains and routes were traced. To do so, a SNP threshold was calculated on the basis of the similarity matrix for all the CNPA strains, calculated *via* the SNP analysis plug-in in BioNumerics™. The epidemiological link provided by the clinical data collected from the patients helped to set the SNP threshold which allowed us to sort the observed pairwise distances in the wgSNP distance matrix into two categories: related and not related. Here sensitivity and specificity are diagnostically equally important and desirable. Therefore, the Youden’s index in conjunction with receiver operating characteristic (ROC) curve was used to indicate the performances of the different SNP cutoff values. The optimal SNP cutoff value was computed using different Youden indicators where sensitivity, accuracy, specificity and Youden’s Index were found to be at their maximum.

## Results and Discussion

### MLST vs. wgMLST

**Figure 1:**
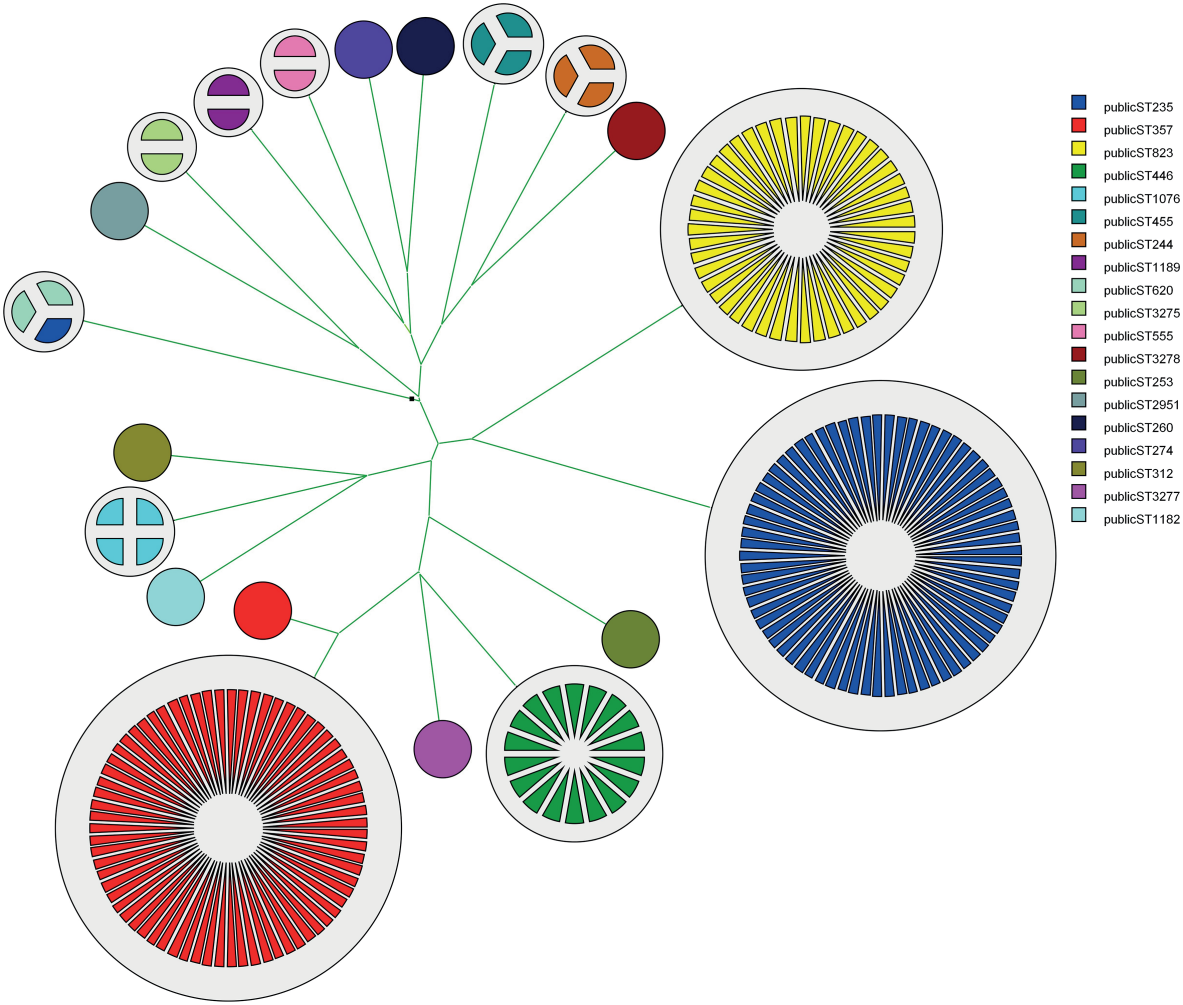
Classical MLST-based phylogenetic tree showing the evolutionary relationship between different CNPA sequence types (ST), each indicated by a different a color and provided with its ST number. Number of partitions in each cluster showing the number of strains in that group. Note that ST357, ST823 and ST235 represent the largest clonal clusters. A similar illustration was presented by Pelegrin et al. (2019).

**Figure 2:**
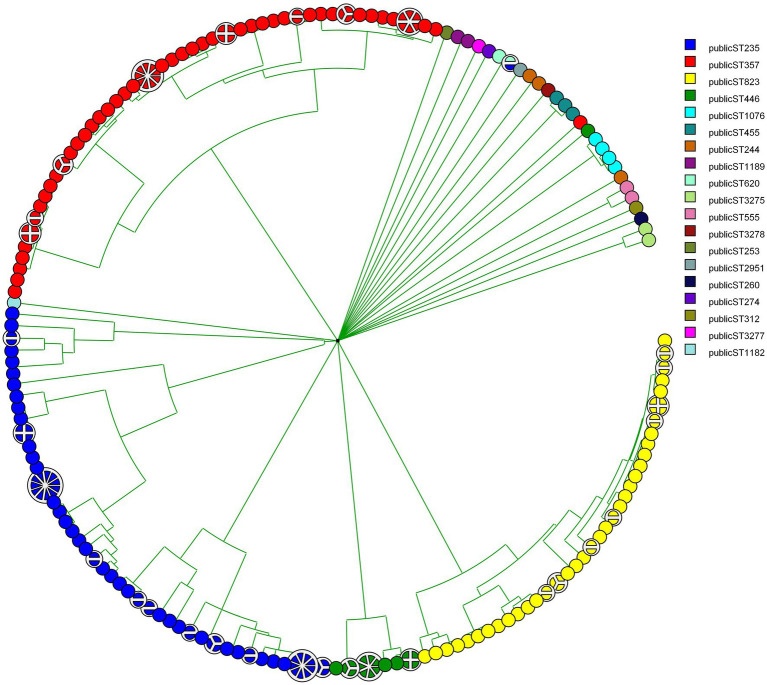
Phylogenetic tree showing CNPA relatedness based on wgMLST. Subgrouping with in each ST (denoted by different colors) is labeled by the original source (patient ID or environmental source) from which these strains have been isolated.

MLST was designed primarily for the purpose of defining global bacterial phylogeny by sequencing internal fragments of seven housekeeping genes (Maiden et al., 1998; Urwin and Maiden, 2003; Jolley et al., 2018). Our set of 237 CNPA strains included 3dominant MLST groups: ST235 (74 isolates) followed by ST357 (72 isolates), and ST823 (47 isolates; Figure 1, shown for reasons of comparison with the genomic methods). Our current MLST results were in complete agreement with those presented by Pelegrin et al. Still, WGS has become the preferred method for studying the molecular epidemiology of bacterial species, clearly providing discriminatory power exceeding that of classical MLST (Kovanen et al., 2014; Pearce et al., 2018). wgMLST analysis allows genome comparisons and recognition of evolutionary subgroups of genetically related isolates within the same classical STs, allowing more refined tracing of the origin of outbreaks and individual infections (Cody et al., 2013; Moura et al., 2016). In the present study we identified subgroups within the major STs (ST235, ST357, ST823), but also within minor STs (Figure 2). Within ST235 the number of allelic variants between isolates ranged between 0 to 200 (11 subgroups) whereas for ST357 it was 0 to 59 (8 subgroups) and for ST823 it was 0 to 39 (8 subgroups). Interestingly, ST235 seemed to contain three different lineages of strains that separated earlier compared to the subgroups detected in the other STs of CNPA. Subgroupings were not specific to patients or clinical sample type, but appeared to be independent and at random (Figure 2). Extended genetic diversity can be seen in the ST235 wgMLST tree as compared to the other STs. However, this might be due to a higher mutation rate in this clade as compared to that of other lineages or indeed, by chance due to an earlier occurrence of diversity within this clade. The main finding here is that wgMLST shows significantly enhanced resolving power as compared to classical MLST. Still, there is excellent concordance between the two methods since there was never any mixing of classical MLST groups at the level of wgMLST groups as was reported by other authors as well (Blanc et al., 2020). A study conducted by Stanton et al. (2020) demonstrated how a cgMLST scheme provided enhanced resolution over traditional MLST, pulsed-field gel electrophoresis (PFGE), and single-nucleotide variant (SNV) assessment to analyze individual outbreaks. That study included core genes those were common to all strains of *P. aeruginosa*. In contrast, wgMLST also covers highly variable elements such as repetitive genes and pseudogenes, depending upon the microbial species studied (Moura et al., 2016). However, clustering of strains based on either the cgMLST or wgMLST can provide a detailed perspective of the taxonomy, epidemiology and evolution of bacterial populations (McNally et al., 2016).

### wgSNP Distribution in CNPA Isolates

**Figure 3:**
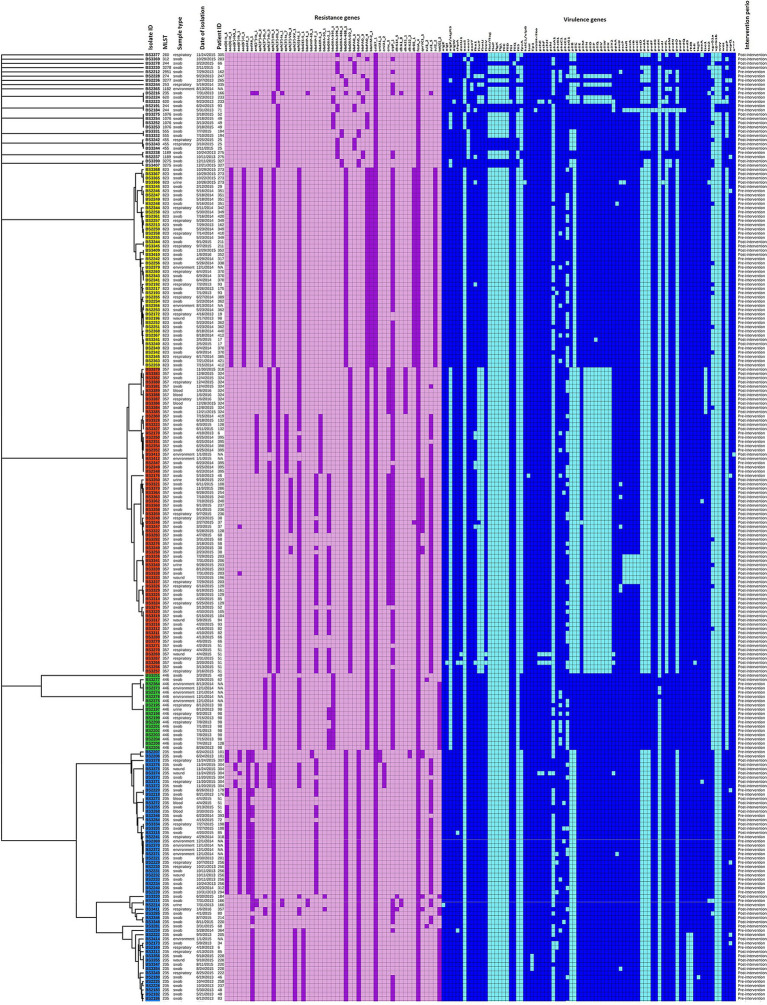
wgSNP-based phylogenetic relationship between CNPA isolates. Major MLST groups are shown with different colors (yellow: ST823; red: ST357 and blue: ST235). Resistance and virulence genes are presented in the form of heat maps with purple and blue color ranges. Epidemiological and clinical data includes isolate ID, MLST, date and source of isolation (patients, environment and sample type) and intervention period are also mentioned along with the tree.

**Figure 4:**
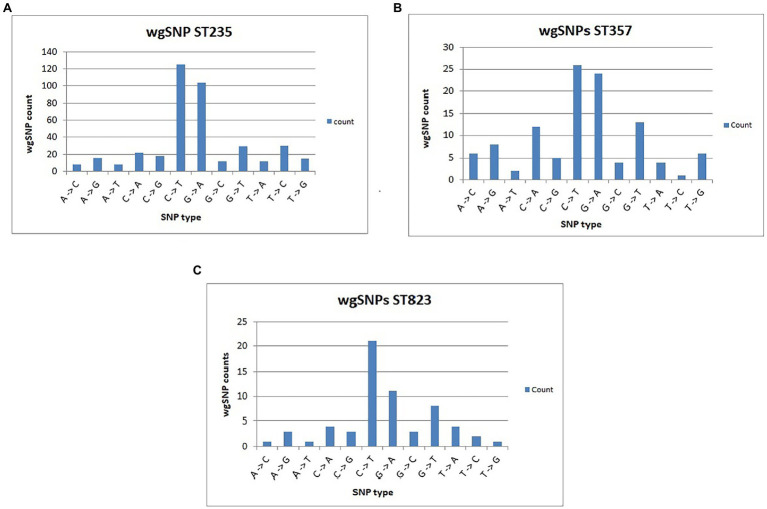
Number of different SNP types with reference to PAO1 strain of *P. aeruginosa* is illustrated for CNPA clone ST235 **(A)**, clone ST357 **(B)** and clone ST823 **(C)**.

wgSNP analysis represents an effective method for characterizing pathogenic bacterial strains and for detecting outbreak events (Bakker et al., 2011; Taylor et al., 2015; Schurch et al., 2018). Recent studies successfully demonstrated the capability of wgSNP-based genotyping to reveal recombination events in *Streptococcus pneumoniae*, *Staphylococcus aureus* and *Cronobacter sakazakii* (Roe et al., 2016; Cowley et al., 2018; Yin and Yau, 2018; Yong et al., 2018). To ensure the accuracy and consistency of SNP-defined outbreak analysis, essential parametric measures such as minimum coverage and distances allowed between SNPs and exclusion of non-informative wgSNPs must be applied (Bakker et al., 2011). In the present study, false SNPs generated due to sequencing or assembling errors were filtered. Retained SNPs including non-informative SNPs were found scattered all across the CNPA genomes. However, after strict filtering of non-informative wgSNPs, SNP counts fell down into the hundreds only (Supplementary Material). Evolutionary relationships between CNPA strains based on strictly filtered informative-wgSNPs were shown in a phylogenetic tree along with their more descriptive epidemiological data and their resistomes and virulomes (Figure 3). It is noteworthy that in the phylogenetic tree, clade ST823 is apparently more homogenous than other STs where identified SNPs were uniformly present in resistome and virulome of all the strains of ST823 (Figure 3). Detailed ST_specific-informative-wgSNP analysis and annotation was performed within only three most dominant CNPA clones (ST235, ST357 and ST823) present in the collection. Previous studies reported ST357 and ST235 clones to be prevalent across the globe and to present a high risk for invasive infection (Treepong et al., 2018; Mihara et al., 2020). The recent emergence of ST823 causing outbreaks in many countries highlighted the importance of evaluating epidemiological trends for clone ST823 (Zowawi et al., 2018; Pelegrin et al., 2019). In the present study a total of number of SNPs (including non-informative SNPs) were ranging from 35,350 in ST235, 34,570 in ST357 and 26,443 in ST823. Informative-wgSNPs ranged from 398 SNPs within ST235 followed by 111 in ST357 to 61 in ST823. All point mutations, their positions and their respective functional annotations are summarized in Supplementary Material. Interesting fact was that out of 12 possible SNP types (based on the availability of the 4 [A, T G and C] bases) only two, C > T and G > A, SNPs were dominant in all the three clones of CNPA (Figures 4A–C). These two SNP types should be further investigated in order to clarify the significance of their predilection in, for instance, genetic adaptation to changes in the environment where *P. aeruginosa* is residing. SNPs were regularly found in transcription regulators and sensor-response regulator hybrids in all the three clones of CNPA strains. Non-informative wgSNPs were those which were present throughout the particular MLST group and very high in numbers. However, the occurrence of informative wgSNPs was different, random and limited to selective number of CNPA strains in the dataset. Common point mutations (excluding non-informative wgSNPs) that were shared by both ST357 and ST235 include those in cytochrome C550 (A > G), the oprD porin (C > T), ABC transporters (T > A), MFS transporters (C > T) and a two-component sensors (G > A). The results presented here underscore that wgSNP typing has a higher resolution than wgMLST and that functional information on individual gene variation can be derived from the data. Of note, a large diversity of antimicrobial resistance genes and virulence genes segregated according to wgSNP analyses (Figure 3). Informative-wgSNP mutations were comparatively low in number and can be analyzed further for a particular mutation type of interest such as in transporter genes, mobile genetic elements and bacteriophages (Supplementary Material). In ST823, no mutation was observed in transporter genes. Still, 7 and 10 SNPs were observed in ST357 and ST235, respectively. Mutations in bacteriophage related genes were only present in ST823. However, mutation associated with mobile genetic elements was not found in any of the three major clones.

### Transmission Dynamics of CNPA Clones

**Figure 5:**
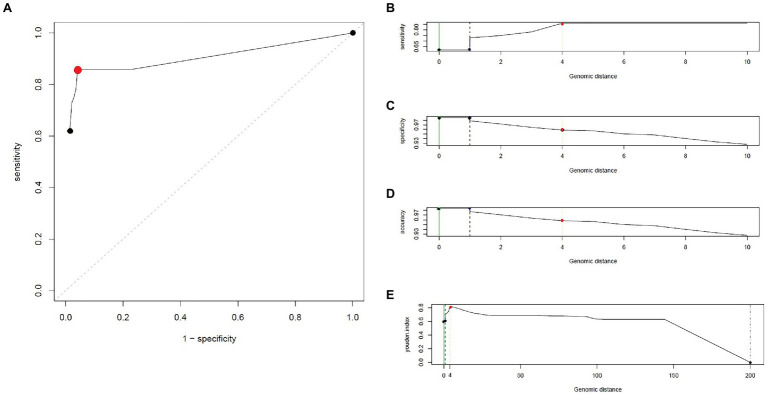
Different Youden indicators calculated using similarity matrix of CNPA strains, generated during SNP analysis. In **(A)**, an ROC curve showing the relationship between clinical sensitivity and specificity for every possible SNP cut-off. Here an optimal point is represented with red colored dot. SNP cutoff values (Genomic distances) are shown on horizontal axis and different statistical parameters or indicators like Sensitivity, Specificity, Youden’s Index and accuracy are shown on vertical axis in **(B-E)**, respectively. Based on all the above mentioned indicators genomic distance of 4 SNPs was chosen as overall optimal SNP cutoff value and is highlighted with red colored dot on each graph.

**Figure 6:**
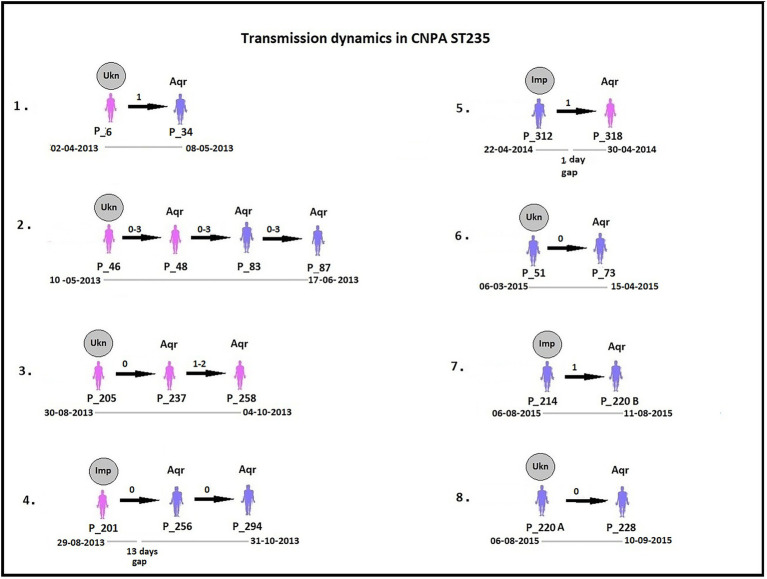
Potential transmissions of CNPA ST235 isolates among the patients (their ID given as “P_”) are shown in the figure. Pink colored patients are from adult-ICU and purple colored patients are from ER-ICU. Strain relatedness in terms of pairwise wgSNP distances (0–4) among the strains is shown on the respective arrow between the two patients harboring strains associated with the transmission event. In some patients, more than one isolate was collected, therefore the pairwise wgSNP distances have a range between the two patients. All these transmissions are arranged in ascending order according to their time of admission to the ICU and their sample collection dates from the year 2013 to 2015. The gray circle above first patient in each transmission event denotes that CNPA strain either imported (if labeled as “Imp”) from outside at the time admission or acquired from unknown (if labeled as “Ukn”) source within the ICU. Other patients in each transmission chain labeled as “Aqr” which means they acquired these clones during hospitalization. The hospitalization time line of the patients is shown below each transmission chain. The gap in the time line depicts that there is a time difference between discharge of one patient and admission of another to the ICU.

**Figure 7:**
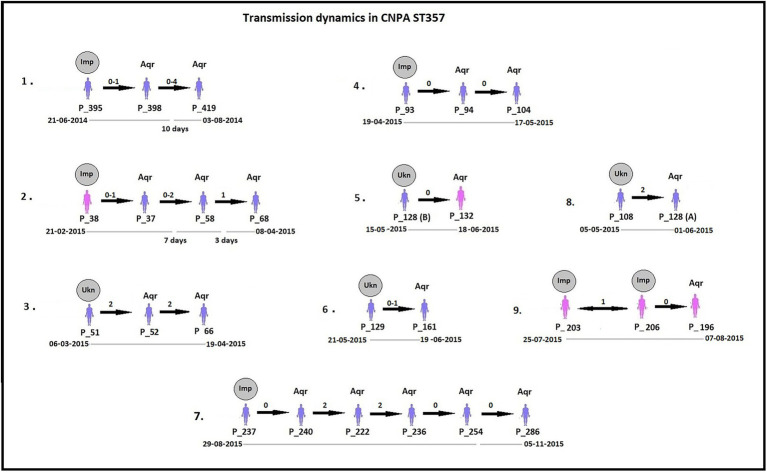
Potential transmissions of CNPA ST357 isolates among the patients are shown. Pink colored patients (their ID given as “P_”) are from adult-ICU and purple colored patients are from ER-ICU. Strain relatedness in terms of pairwise wgSNP distances (0–4) among the strains is shown on the respective arrow between the two patients harboring strains associated with the transmission event. In some patients, more than one isolate was collected, therefore the pairwise wgSNP distances have a range between the two patients. All these transmissions are arranged in ascending order according to their time of admission to the ICU and their sample collection date from the year 2013 to 2015. The gray circle above first patient in each transmission event denotes that CNPA strain either imported (if labeled as “Imp”) from outside at the time admission or acquired from unknown (if labeled as “Ukn”) source within the ICU. Other patients in each transmission chain labeled “Aqr” which means they acquired these clones during hospitalization. Hospitalization time line of the patients is shown below each transmission chain. The gap in the time line depicts that there is a time difference between discharge of one patient and admission of another to the ICU. 6^th^ Transmission chain shows an unusual situation where two patients (203 and 206) possibly the part of a transmission event but both of them were already imported with ST235 strain. Therefore the probability of transmission could be to or from P_203 or P_206 during their overlapping days of stay in the ICU.

**Figure 8:**
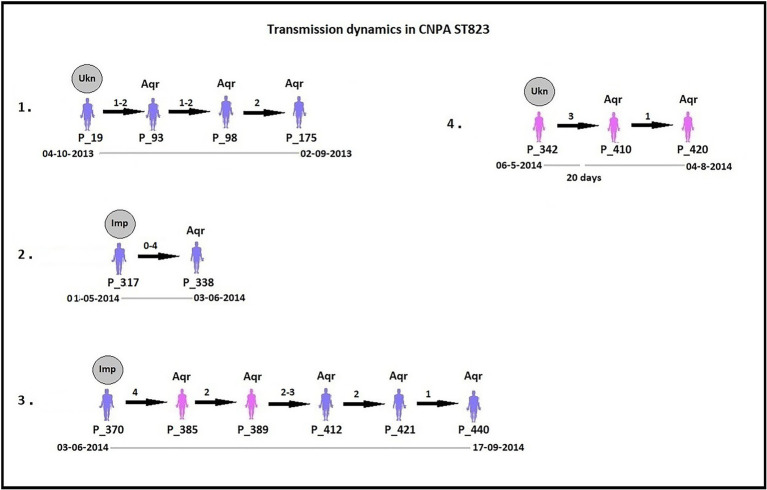
Potential transmissions of CNPA ST823 isolates among the patients (their ID given as “P_”). Pink colored patients are from adult-ICU and purple colored patients are from ER-ICU. Strain relatedness in terms of pairwise wgSNP distances (0–4) among the strains is shown on the respective arrow between the two patients harboring strains associated with the transmission event. In some patients, more than one isolate was collected, therefore the pairwise wgSNP distances have a range between the two patients. All these transmissions are arranged in ascending order according to their time of admission to the ICU and their sample collection date from the year 2013 to 2015. The gray circle above first patient in each transmission event denotes that CNPA strain either imported (if labeled as “Imp”) from outside at the time admission or acquired from unknown (if labeled as “Ukn”) source within the ICU. Other patients in each transmission chain labeled as “Aqr” which means they acquired these clones during hospitalization. Hospitalization time line of the patients is shown below each transmission chain. The gap in the time line depicts that there is a time difference between discharge of one patient and admission of another to the ICU.

By analyzing SNPs in CNPA isolates within the three major ST groups, we have determined the likelihood of their transmission from one patient to another in the setting of the two ICUs in the Indonesian hospital where the clinical part of our study took place. In this study, an optimal SNP cutoff value of ≤4 SNPs was calculated using pairwise distance matrix of informative-wgSNPs for the CNPA dataset using Youden indicators (Figure 5). The Youden index provides the optimal cutoff point with maximum differentiating ability and effectiveness of a diagnostic biomarker when equal weight is given to the sensitivity and specificity (Youden, 1950; Ruopp et al., 2008). Many previous studies demonstrated the utility of Youden index as a reliable method to calculate optimal cut-off value of a biomarker such as SNP (Pelegrin et al., 2019; Oh et al., 2020). In this study the Youden’s indicator was at a maximum for a threshold of 4 SNP (sensitivity 0.86, specificity 0.96, accuracy 0.96, Youden index 0.82). As reported, individual strains of *P. aeruginosa* can be considered genetically indistinguishable if their pairwise SNPs differ less than 3–5 SNPs (Quick et al., 2014; Parcell et al., 2018; Pelegrin et al., 2019). Previously, Pelegrin et al. (2019) reported 50 strain acquisition events in this cohort of ICU patients on the basis of genomic proximity (at threshold <5 SNPs) and clues from clinical data. Using the optimal SNP cutoff of ≤4 SNPs, we have now re-traced those acquisition events in more detail and further elucidated the chains of transmission based on pairwise SNP distances among the strains. According to the clinical metadata collected from the hospital, patients were tested for CNPA strain colonization. If tested positive then the patient was labeled as “Imported (Imp).” In any other instant the patient was labeled as “Acquired (Aqr).” Some isolates collected from the same patient (not from the previously collected sample of the same patient) were later identified as different clones. Therefore, patients who were colonized by multiple strains were studied and considered while tracing the transmission events. Genetically indistinguishable strains isolated from patients without overlapping hospitalization periods were considered as possibly originating from the same source only if their pairwise distance of informative-wgSNPs was ≤4 SNPs and the time difference between the hospitalizations, i.e., between the departure of one patient and the admission of another, was not more than 16 months (Kramer et al., 2006). These events were defined as healthcare-associated transmissions and were traced on the basis of strictly filtered pairwise distances of ST-specific-informative-wgSNPs (excluding non-informative wgSNPs) only. In the group of 36 patients harboring a ST235 strain, 11 were already carrying “their” strain at the time of admission to the ICU also called as Imported strain (Imp). The remaining patients acquired (Aqr) a ST235 strain during their hospitalization period. While tracing the probable transmission where a transmission event occurred first with a patient labeled as “Aqr” in the metadata was actually from an unknown source with in the hospital itself. Therefore, each transmission chain starting with the patient harboring a strain from an unknown source (e.g., the environment) was labeled as “Unknown (Ukn)” (Figures 6–8). The number of identified pairwise distances of ST-specific-informative-wgSNPs within each transmission chain illustrated the strain relatedness under the threshold of 4 informative-wgSNPs (Supplementary Material). Also the overlapping hospitalization period was shown with a straight line in each transmission chains along with the dates, whereas a small considerable gap in this time line showed the time gap between one patient’s discharge and admission of the other. On the basis of genomic identity among ST235 (pairwise distances of ST-specific-informative-wgSNPs) and the time of patient’s admission in the ICU, we found 8 possible chains of transmission including 3 within the ER-ICU and one in the adult-ICU; the remaining 4 events were probably inter-ICU transmissions (Figure 6). In ST357, 34 patients (of which 12 were positive on admission) were involved in 9 transmission chains (Figure 7), and five potential chains of transmission were detected among 25 patients harboring ST823 clones (of whom eight carried the strain at the time of their admission). In ST357 6 events were within ER-ICU, 2 were associated with Adult-ICU and 1 remaining was belong to inter-ICU transmission. In the latter group ST823 only two transmission chains occurred within the ER-ICU, one within the adult-ICU and one involving both ICUs (Figure 8). Thus, where Pelegrin et al. (2019) only presented qualitative data regarding possible transmissions and the overall number of acquisition events, we here reveal possible chains of transmission of CNPA strains between patients. Importantly, wgSNP analysis also allows for a better characterization of strains already carried by patients at the time of their hospitalization. This study contains a few isolates collected from the environment, none of which was found to be correlated with overlapping hospitalization and within the limits of informative-wgSNP cutoff values for the patient-derived strains. However a very small gap between discharge and admission of the two patients harboring isogenic strains suggests the transmission could take place through the environment of health care setting. Thus we suggest that this study illustrates the possible transmission events even for those with limited clinical information available. However, to achieve further certainty of these events, additional environmental sampling is suggested.

## Conclusion

We here show that in comparison with more classical methods wgMLST and wgSNP analyses provide enhanced resolution for the epidemiological typing of strains of *P. aeruginosa*. The use of WGS data will provide typing schemes of high discrimination capacity and, depending on the density of sampling, allow for more precise mapping of the flow of *P. aeruginosa* going through susceptible patient cohorts. This should in the end help improve infection prevention.

## Data Availability Statement

The original contributions presented in the study are included in the article/Supplementary Material, further inquiries can be directed to the corresponding author.

## Author Contributions

AB and MG contributed to study design. MG did all the analysis and wrote the initial draft of the manuscript. AB did major manuscript editing. AP, MJ, YS, CK, HV, and JS helped in editing the manuscript upto the final version. All authors contributed to the article and approved the submitted version.

## Funding

This research was supported and funded by bioMérieux, France and the European Union’s Horizon 2020 research and innovation program entitled Viral and Bacterial Adhesin Network Training (ViBrANT) under Marie Skłodowska-Curie Grant Agreement no. 765042.

## Conflict of Interest

During this study MG, MJ and AB were employees of bioMérieux, a company designing, developing, and marketing tests in the domain of infectious diseases. The company was not involved in the design of the current study and the opinions expressed are those of the authors and may be different from formal company opinions and policies.

## Publisher’s Note

All claims expressed in this article are solely those of the authors and do not necessarily represent those of their affiliated organizations, or those of the publisher, the editors and the reviewers. Any product that may be evaluated in this article, or claim that may be made by its manufacturer, is not guaranteed or endorsed by the publisher.

## Footnotes

^1^ http://pubmlst.org

^2^ www.applied-maths.com/applications/wgmlst

^3^ www.applied-maths.com/applications/whole-genome-snp-analysis

^4^ https://github.com/tseemann/abricate
